# Aflatoxin B_1_ contamination in maize in Europe increases due to climate change

**DOI:** 10.1038/srep24328

**Published:** 2016-04-12

**Authors:** P. Battilani, P. Toscano, H. J. Van der Fels-Klerx, A. Moretti, M. Camardo Leggieri, C. Brera, A. Rortais, T. Goumperis, T. Robinson

**Affiliations:** 1 Università Cattolica del S. Cuore di Piacenza Faculty of Agricultural, Food and Environmental Sciences, Department of Sustainable Crop Production, via Emilia Parmense 84, 29100 Piacenza, Italy; 2 National Research Council - Institute of Biometeorology (CNR-IBIMET), Via Caproni 8, 50145 Florence, Italy; 3 RIKILT Wageningen UR, Department of Toxicology, Bio-assays & Novel Foods, Akkermaalsbos 2, NL-6708 WB, Wageningen, The Netherlands; 4 Institute of Sciences of Food Productions, CNR, Via Amendola 122/O, 70126, Bari, Italy; 5 Istituto Superiore di Sanità, Veterinary Public Health and Food Safety Department, Viale Regina Elena 299, 00161 Rome, Italy; 6 European Food Safety Authority, Scientific Committee and Emerging Risks Unit, Via Carlo Magno 1A, 43126 Parma, Italy

## Abstract

Climate change has been reported as a driver for emerging food and feed safety issues worldwide and its expected impact on the presence of mycotoxins in food and feed is of great concern. Aflatoxins have the highest acute and chronic toxicity of all mycotoxins; hence, the maximal concentration in agricultural food and feed products and their commodities is regulated worldwide. The possible change in patterns of aflatoxin occurrence in crops due to climate change is a matter of concern that may require anticipatory actions. The aim of this study was to predict aflatoxin contamination in maize and wheat crops, within the next 100 years, under a +2 °C and +5 °C climate change scenario, applying a modelling approach. Europe was virtually covered by a net, 50 × 50 km grids, identifying 2254 meshes with a central point each. Climate data were generated for each point, linked to predictive models and predictions were run consequently. Aflatoxin B_1_ is predicted to become a food safety issue in maize in Europe, especially in the +2 °C scenario, the most probable scenario of climate change expected for the next years. These results represent a supporting tool to reinforce aflatoxin management and to prevent human and animal exposure.

Climate change has been reported as a driver for emerging food and feed safety issues worldwide^1^,^2^,^3^,^4^.The expected impact of climate change on the presence of mycotoxins in food and feed is of great concern. These fungal metabolites are important causes of chronic toxicity from exposure via food^5^,^6^; in particular, aflatoxins, which have the highest acute and chronic toxicity of all mycotoxins^7^. Hence, the maximal concentration of aflatoxins in agricultural food and feed products and their commodities is regulated worldwide, with specific restrictions in Europe (Commission Regulation EU/574/2011, 2006/1881/EC and amendments). The possible change in patterns of aflatoxin occurrence in food and feed crops due to climate change is a matter of concern that may require anticipatory actions.

Crop growth and its interaction with beneficiary and pathogenic and/or toxigenic microorganisms vary from year to year, mainly depending on local weather, making the agricultural sector particularly exposed to climate change^8^. The topic is of great economic and societal interest both for the quantitative and qualitative effects on crop yield and the impact on the occurrence of mycotoxins^9^.

Around nine million hectares of maize and 26 million hectares of common wheat are yearly grown in Europe, and cereals in general contribute to approximately 30% of the human diet of industrialised countries (data from FAOStat http://faostat3.fao.org/home/E), as well as to roughly 50% of the animal feed in Europe (European Commission, Agricultural and Rural Development, Short term outlook, 2015). Therefore, any problem related to food and feed crops is of great economic and health concern.

The most toxic mycotoxins are aflatoxins, which can occur in host crops infected by some species of *Aspergillus*. Aflatoxins are genotoxic, carcinogenic and immunosuppressive substances, and cause both acute and chronic toxicity. Related health problems are difficult to diagnose, mainly due to cryptic, long-term and chronic exposures. However, as recently shown and recognized by the Kenyan government, in 2004 and 2005, hundreds of human death cases can be ascribed to the consumption of aflatoxin contaminated maize products^10^.

Aflatoxins are reported in several agricultural crops, mainly maize, peanuts, pistachio nuts and cottonseeds^11^. Maize contamination is of very high concern since this crop plays a main role both in food and feed supply worldwide. Until a few years ago, aflatoxins have not been signalled as a matter of concern for primary production in Europe^12^. However, the years 2003 and 2012 must be mentioned, for Italy and South Europe, respectively, because of the alarming contamination in maize^13^,^14^,^15^. Very low aflatoxin contamination was reported in the few studies published on wheat^16^,^17^,^18^, suggesting that, so far, aflatoxins are of minor concern in this crop and its derived products.

*Aspergillus flavus*, the key fungus for aflatoxin production, is well adapted to warm and dry weather conditions^19^. Crops from tropical and/or sub-tropical areas are affected more frequently and severely by aflatoxin contamination, but temperate areas could be of increasing importance due to climate change. Exploring scenarios for this emerging hazard in temperate areas in order to predict future trends and anticipate potential control options is, therefore, crucial. To face this challenge, a modelling approach was considered in this study.

## Results

### Predictive models and climate change data

AFLA-maize, a mechanistic model, was applied to predict *A. flavus* growth and aflatoxin production in maize, using weather data as input^20^. AFLA-maize was also linked to a crop phenology prediction module, based on temperature sums, with a focus on the crucial stages of flowering and ripening, or date of harvest. Two climate change scenarios, +2 °C and +5 °C above pre-industrial levels, which take into account whether or not mitigation strategies for climate change are applied, in addition to the present (baseline) scenario, were considered.

For each scenario, 100 years of simulated daily weather data (minimum and maximum temperatures, rainfall and solar radiation) were obtained for each of the 2254 georeferenced (central) grid points, with grids spaced 50 × 50 km, over the European domain. These data were used as input for running both the crop phenology model and the AFLA-maize model. Output of the crop phenology model, i.e. predicted flowering and harvest dates, was also used as input for AFLA-maize. The final output included aflatoxin hazard indexes (AFI) for each of the mentioned grid point (2254 points) throughout Europe, per simulation run (100 years) and scenario (present, + 2 °C and +5 °C climate change scenarios ; see supplementary material for details). AFI = 0 when the host crop: i) cannot be grown in the grid point due to unsuitable meteorological conditions, or ii) its susceptibility to *A. flavus* never occur or never match. When AFI > 0, fungus-host interactions are possible as well as the production of aflatoxins. Referring to the current legal limits in Europe (Commission Regulations 574/2011 and 1881/2006 with amendments), the threshold of 5 μg of aflatoxin per kg of raw maize kernels was considered consistent with AFI ≥ 95.

Data on *A. flavus*-wheat interactions and predictive models were not available from the literature. Therefore, AFLA-wheat, a modified version of the AFLA-maize model, was developed and run using the same meteorological input data as with the AFLA-maize model (See supplementary material for details). An AFI threshold for high probability of wheat contamination was not available, due to the lack of data on grain contamination. Therefore, an AFI ≥ 38 was considered to coincide with the legal limit for aflatoxin B_1_ in wheat of 2 μg of aflatoxin per kg of raw grains (Commission Regulation 1881/ 2006 and amendments).

### Aflatoxin prediction

The model output, i.e., the AFI computed for maize and wheat, was summarised representing the 2254 grid points in Europe, 100 simulated years, and the three scenarios (Table 1).

In the present scenario, for maize, mean AFI computed over all sites was 38, the percentage of sites with mean AFI > 0 was 39%, but only in 20% of the sites mean AF contamination was predicted to be above the current legal limit in Europe (AFI > 95). In the +2 °C and the +5 °C scenarios, mean AFI (over all sites) was 73 and 95, respectively. Mean AFI increased by 92% and 149% when moving from the present scenario to the +2 °C and +5 °C scenarios, respectively, and 39% and 54% of the sites with AFI > 95.

**Figure 1:**
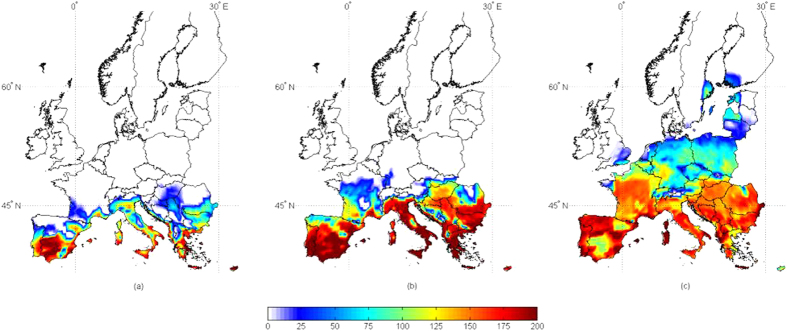
Risk maps for aflatoxin contamination in maize at harvest in 3 different climate scenarios, present, +2 °C, +5 °C. Mean daily data used as input result from 100-year run of the predictive model AFLA-maize in 2254 geo-referenced points throughout Europe, in the 3 scenarios. The scale 0–200 refers to the aflatoxin risk index (AFI), output from the predictive model; increasing the (present (a), +2°C (b), +5°C (c)) number, the risk of contamination increases. Maps generated using Mathworks, Matlab. Computer Program, 2012 http://it.mathworks.com/.

**Figure 2:**
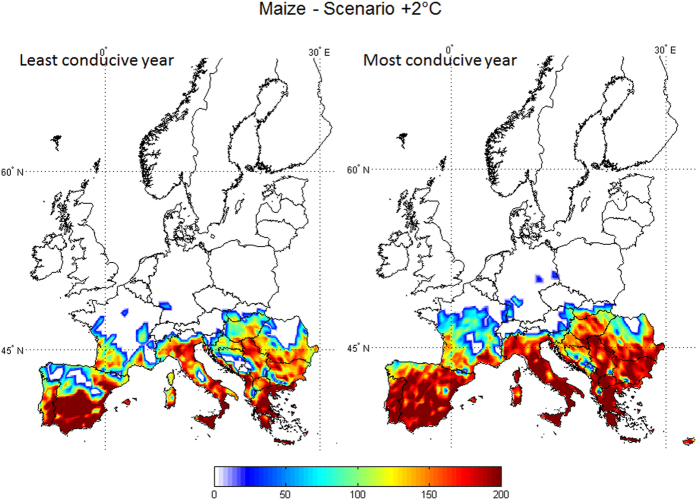
Risk maps for aflatoxin contamination in maize at harvest in the +2 °C climate scenario. Mean daily data used as input result from 100-year run of the predictive model AFLA-maize in 2254 geo-referenced points throughout Europe. The least (a) and the most (b) conducive years were represented. The scale 0–200 refers to the aflatoxin risk index (AFI), output from the predictive model; increasing the number, the risk of contamination increases. Maps generated using Mathworks, Matlab. Computer Program, 2012 http://it.mathworks.com/.

Maps were drawn using geo-referenced grid points to highlight the predicted future aflatoxin contamination in Europe based on predicted AFI (Fig. 1). In the present scenario, the area with possible aflatoxin production in maize (AFI > 0) is mainly below the 45° North latitude, because of the limiting conditions for *A. flavus* growth and aflatoxin production at higher latitudes. Increasing temperature clearly enlarges the area of interest for maize. In the +5 °C scenario, maize farming is predicted in latitudes as high as 60° North, a much more northern geographic distribution than the current maize growing area. In both climate change scenarios, the most concerned areas with an increase of aflatoxin contamination were: Eastern Europe, Balkan Peninsula and the Mediterranean regions. However, the highest mean AFI values were found under the +2 °C scenario, frequently in the range of 160–200. Aflatoxin contamination above the legal limit are expected to become more frequent in the future, based on our results. This prediction is further confirmed by results shown in Fig. 2, presenting the minimum and maximum probabilities of aflatoxin contamination in the 100 simulated years in the +2 °C scenario. The south of Europe has a high chance of becoming a risk area for aflatoxin contamination in maize, even in the least conducive year (i.e. with the lowest AFI value). Under the +2 °C scenario, the growing area of maize is expected to be almost the same as in the present scenario and an advance in flowering and harvesting dates is limited to 5 and 10 days, respectively (See supplementary material for details). Therefore, in this context, the agricultural management practices are not expected to change significantly, which makes predictions, obtained using only weather data as input, even more robust. The reliability of results is further supported by the confirmed accuracy of model predictions, irrespective of extreme weather conditions, and its sensitivity to data input, especially temperature.

Regarding wheat, in the present scenario, mean AFI was 0.7. According to the model predictions, over 90% of the sites may allow *A. flavus* growth (AFI > 0), although with very low AFI values. Mean AFI increased by 60% and more than doubled in the +2 °C and +5 °C scenarios, respectively (Table 1), but the probability of aflatoxin contamination in wheat is so low that it can be considered irrelevant.

**Figure 3:**
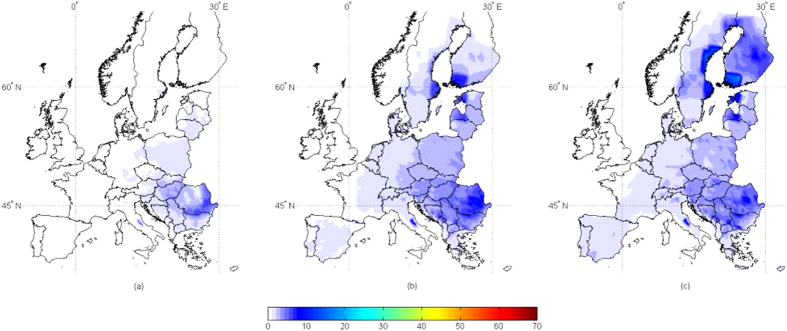
Risk maps for aflatoxin contamination in wheat at harvest in 3 different climate scenarios, present, +2 °C, +5 °C. Mean daily data used as input result from 100-year run of the predictive model AFLA-maize in 2254 geo-referenced points throughout Europe, in the 3 scenarios (present (a), +2°C (b), +5°C (c)). The scale 0–200 refers to the aflatoxin risk index (AFI), output from the predictive model; increasing the number, the risk of contamination increases. Maps generated using Mathworks, Matlab. Computer Program, 2012 http://it.mathworks.com/.

**Figure 4:**
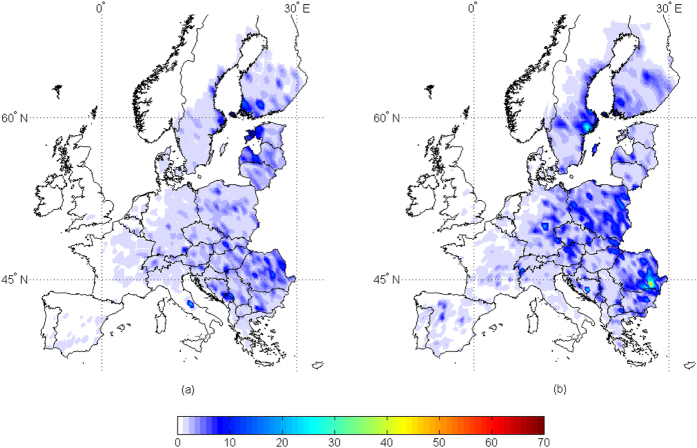
Risk maps for aflatoxin contamination in wheat at harvest in the +2 °C climate scenario. Mean daily data used as input result from 100-year run of the predictive model AFLA-maize in 2254 geo-referenced points throughout Europe. The least (a) and the most (b) conducive years were represented. The scale 0–200 refers to the aflatoxin risk index (AFI), output from the predictive model; increasing the number, the risk of contamination increases. Maps generated using Mathworks, Matlab. Computer Program, 2012 http://it.mathworks.com/.

When AFI > 5 is considered, only 1% of the sites have a contamination above this threshold in the present scenario, increased to 3.5% and 4.4% in the +2 °C and +5 °C scenarios, respectively (Figs 3 and 4). For all three scenarios, an increase in the mean AFI was found in several areas, but mainly in central Italy, Eastern Europe and close to the Baltic Sea, always with low probabilities of contamination. This confirms findings reported so far^16^,^17^,^18^,^21^.

Considering variation between years, the lowest and the highest means of AFI computed for maize are, respectively, 0.5 and 0.9 for the present scenario, 0.83 and 1.49 for the +2 °C scenario, and 1.03 and 2.06 for the +5 °C scenario. Therefore, a decrease of around 20% and an increase of around 34% with respect to the means were determined in the least and most conducive year, respectively. The variation between years is comparable in the +2 °C scenario, with −22% and +38%, and in the +5 °C scenario (−28% and +43%).

Based on this modelling approach to predict future aflatoxin contamination in maize grown in Europe, the +2 °C climate change scenario^22^, currently considered the most reliable for the next 100 years^23^, resulted into a significant increase in the estimated aflatoxin contamination in all maize growing areas, whereas the predicted impact on contamination in wheat was negligible.

### Impact on European countries

In the current climatic conditions, European countries in which maize cultivation is common, i.e. in Romania, France, Hungary and north-east Italy (in total accounting for 60% of the total production in 2013 for the 28 EU Member States, FAOStat, 2013), show a low probability of aflatoxin occurrence. An increase from a low to a medium probability of aflatoxin contamination is found under the +2 °C scenario. Critical and high AFI are predicted in some areas of southern European countries (i.e. in Greece, southern Italy, Bulgaria and Albania), in which maize production is marginal, but important for the local population. The +5 °C scenario leads to a larger European domain exposed to aflatoxin contamination. However, despite these figures, an increase in AFI in southern Europe is of limited concern since conditions are less suitable for *A. flavus* growth. Northern European countries are currently in the safe zone regarding aflatoxin contamination, but they may face new climatic conditions leading to a new agro-socio-economic context.

## Discussion

The future scenario of +2 °C hypothesized for Europe is realistic^23^ and could change several plant-pathogen interactions in the whole continent^24^. In particular, due to climate change, aflatoxin contamination of maize kernels will be an important concern for food and feed safety and security. A reduction of safe maize production is predicted by this study, and was also recently observed^14^,^15^. This would reduce availability of maize for both food and particularly feed uses. Contaminated crops will have a reduced economic value, as they need to be diverted to other uses such as biofuel generation. This could lead to an end of maize production in current areas as being uneconomical. In addition, the enlargement of aflatoxin risk zones could increase human and animal population chronic exposures to these mycotoxins.

Occurrence of aflatoxins in food and feed is therefore a “hot issue” in Europe for policy makers. The first regulation of the European Commission was adopted in 2001 setting the maximum limits for foodstuffs (Commission Regulation 2001/466/EC) and in 1974 for animal feeding stuffs (Council Directive 74/63/EEC of 17 December 1973) and several amendments followed. In general, official control measures contribute to the global effort to reduce risks for human and animals from exposure to aflatoxins by food and feed intake. However, specific action plans also need to be directed to the production chain.

We consider aflatoxin contamination as a main emerging issue related to maize grown in Europe. We suggest the modelling approach as a supporting tool to reinforce aflatoxin management in maize, to prevent human and animal exposure and related health risks^25^.

Indeed, new strategies supported by predictions should be adopted^25^, such as biological control using atoxigenic *A. flavus* strains, able to displace the toxigenic populations of the fungus, as largely applied in risk areas in the USA and Africa^26^,^27^.

From a risk assessment perspective, the aflatoxin risk maps produced in this study could be used as a communication tool for stakeholders, especially for farmers and stockbreeders. Moreover, the maps could also be used as a management tool to highlight aflatoxin contamination risk areas in order to prioritize aflatoxin control and intervention strategies.

The approach followed in this study is transferable to a global level and can significantly support strategic decisions especially in high risk areas, frequently placed in emerging countries with limited resources, to contribute in reducing local population exposure to aflatoxins and therefore in improving public and animal health.

A similar approach could be applied to other crop-mycotoxin combinations. In the perspective of a dramatic switch of the toxigenic fungal population profiles, as recorded by several scientists over a wide range of geographical areas and in various types of crops, such a modelling approach could become a powerful tool for a more efficient management of mycotoxin contamination worldwide.

## Methods Summary

### Climate change data

Global Change Model (GCM) (IPCC, 2007) was used in this study to generate climate change scenarios. An empirical downscaling procedure was applied, due to the coarse resolution of GCM climate data, in order to reproduce the future European climate at a regional scale using a weather simulator (LARS WG^28^). The results of Hardley Centre coupled Model, version 3 (HadCM3 GCM) for A2 scenario (time slices were centred over the 2030–2059 and 2070–2099 time periods, for +2 °C and +5 °C,respectively) were used to derive the forcing factors to be used in the downscaling procedure. Following the approach proposed by Moriondo, *et al.*^29^, the observed daily data of 2254 grid points (Tmin, Tmax, rainfall and radiation) for the period 1975–2005, spaced 50 × 50 km (data from MARS project http://mars.jrc.ec.europa.eu/) were used for the local calibration of the stochastic weather generator. These were computed for 304 selected GCM (Global Circulation Model) grid points over the domain, as monthly average differences of Tmin, Tmax, rainfall and solar radiation between the future and the reference period. Then, forcing factors calculated for each GCM grid were applied in the downscaling procedure to perturb the relevant climatology of the observed dataset generating stochastically 100 years of daily data for each 50 × 50 km grid point corresponding to an average increase of +2 °C (Scenario +2 °C) and +5 °C (Scenario +5°).

The results of the statistical downscaling procedure related to each grid point were then used to feed the crop phenology prediction model and AFLA-maize, the predictive model for aflatoxin risk^20^.

### Predictive models

The mechanistic model, AFLA-maize^20^, was applied to predict aflatoxin contamination in maize during the growing season, at the European level. Briefly, the mechanistic model is based on the infection cycle of *A. flavus* in maize, it is weather-driven and predictions are given on a daily basis from silk emergence to harvest. Hourly data of air temperature, relative humidity and rain were used as model data input, and they served as input to estimate crop phenology (in the crop phenology module) and fungal activity (in AFLA-maize). The crop phenology module used regression equations to estimate the shift in crop development in the future climate. Besides the climate data, it used databases with information on the cultivation of maize as well as the related temperature requirements, per grid, in Europe. Temperature requirements (JRC data) related to Degree Day (DD, the summation of daily temperature) needed to reach the cropping stages of silk emergence, the time of crop susceptibility to *A. flavus* infection, and harvest time were defined. Model output is the aflatoxin index (AFI), which results from the summation of daily outputs. The index has been associated to a probability of overcoming the threshold of 5 μg of toxin per kg of kernels at harvest applying a logistic equation between AFI and six years of field data of aflatoxin content in maize collected in Italy.

Climate change data are generated on a daily scale; therefore, the model AFLA-maize was adapted accordingly. A new logistic regression was developed to associate AFI, based on daily climate data, to aflatoxin contamination in the field, using the same six-year data as previously mentioned, and AFI > 95 was the resulting threshold value associated to the 50% probability of contamination above 5 μg of aflatoxin per kg of kernels.

Sensitivity analysis was applied. Input data for AFLA-maize were manipulated; Temperature was changed from −6 °C to +6 °C, Relative Humidity and Number of rainy days from −30% to +30% and following AFI changes determined (See supplementary material for details).

Accuracy of model prediction changing data input was estimated based on the correlation coefficient (R^2^) using bootstrap samples (10000 iterations^30^). Air temperature, relative humidity and rainy days were used as as regressors in a Linear Regression Model to reconstruct the variability of AFI simulated for the present climate scenario and to estimate uncertainty associated with AFLA-maize model when extrapolated to climate change scenarios. Eight years were selected, characterized by the largest variations compared to year representing the mean values (See supplementary material for details).

A modified version of AFLA-maize, called AFLA-wheat, was used for predictions in wheat. Starting point of simulation in the crop phenology module was 1 January, and temperature requirements related to DD needed to reach the cropping stages, like flowering and harvest time were defined. In this case, with very limited field data on aflatoxin contamination in wheat, no probability of overcoming the threshold could be associated to the AFI value obtained. Therefore, comparing the legal limit for aflatoxin B_1_ in maize and wheat (5 *versus* 2 μg of aflatoxin per kg of kernels), AFI > 38 was arbitrary associated to the 50% probability of contamination above the threshold.

### Data analysis and mapping

For each scenario (present, +2 °C and +5 °C), AFI values, computed for 100 years, were grouped for each grid point (2254 grid points) and basic statistics (average, standard deviation and standard error) were calculated for each grid point.

Mean risk indices were presented as a spatial risk distribution for maize and wheat for each of the three scenarios. The iterations with the highest and the lowest risk prediction were also drawn.

## Additional Information

**How to cite this article**: Battilani, P. *et al.* Aflatoxin B_1_ contamination in maize in Europe increases due to climate change. *Sci. Rep.* **6**, 24328; doi: 10.1038/srep24328 (2016).

## Supplementary Material

Supplementary Information

## Figures and Tables

**Table 1:** Summary statistics for estimated aflatoxin risk index (AFI) values in maize and wheat in Europe for 100 years, in the present, +2 °C and +5 °C climate change scenarios.

	Average_present	Average_+2 °C	Average_+5 °C
Maize			
Mean (*Median*)*	38.20 (0.00)	73.25 (1.23)	95.09 (108.49)
Conf. interval§	35.54–40.92	69.63–76.82	92.18–98.06
Range	(0.00–225.18)	(0.00–235.01)	(0.00–221.54)
St. dev. Mean#	9.404	14.998	25.297
Max St. dev. Site+	44.821	69.987	57.176
Wheat			
Mean (*Median*)*	0.67 (0.20)	1.08 (0.32)	1.44 (0.68)
Conf. interval§	0.63–0.72	1.01–1.14	1.36–1.52
Range	(0.00–7.01)	(0.00–9.60)	(0.00–18.39)
St. dev. Mean#	0.753	1.229	1.667
Max St. dev. Site+	10.305	15.412	17.817

Mean with related standard deviation, maximum and minimum risk indexes computed for 100 runs, simulating 100 years, for 2254 grid points in Europe are reported.

^*^Values comes from 225.400 data (2254 grid point per 100 years).

^§^Confidence interval of mean AFI, P=95%.

^#^Standard deviation of mean AFI.

^+^Values comes from 100 data (100 years).
