# Supplementary material for: Aflatoxin B1 contamination in maize in Europe increases due to climate change
Source: Sci Rep. 2016 Apr 12;6:24328. doi: 10.1038/srep24328 (PMC4828719; doi:10.1038/srep24328)
Supplement: Supplementary Information [file srep24328-s1.pdf]

Aflatoxin B_1 contamination in maize in Europe increases due to climate change

[1*]Battilani P., [2]Toscano P., [3]Van der Fels-Klerx H.J., [4]Moretti A., [1]Camardo Leggieri M., [5]Brera C., [6]Rortais A., [6]Goumperis T., [6]Robinson T.

[1]Università Cattolica del S. Cuore di Piacenza Faculty of Agricultural, Food and Environmental Sciences, Department of Sustainable Crop Production, via Emilia Parmense 84, 29100 Piacenza, Italy.

[2]National Research Council - Institute of Biometeorology (CNR-IBIMET), Via Caproni 8, 50145 Florence, Italy

[3]RIKILT Wageningen UR, Department of Toxicology, Bio-assays & Novel Foods , Akkermaalsbos 2, NL-6708 WB, Wageningen, The Netherlands.

[4]Institute of Sciences of Food Productions, CNR, Via Amendola 122/O, 70126, Bari, Italy

[5]Istituto Superiore di Sanità, Veterinary Public Health and Food Safety Department, Viale Regina Elena 299, 00161 Rome, Italy

[6]European Food Safety Authority, Scientific Committee and Emerging Risks Unit, Via Carlo Magno 1A, 43126 Parma, Italy

paola.battilani@unicatt.it; Phone: +39 0523 599254

Supplementary material

Sensitivity analysis

Within the baseline scenario (Present Scenario) the simulated 50-year was selected as one with the closest temperature and precipitation to the present condition for a sensitivity analysis.

To analyze the impact of extreme possible changes of +2°C and +5°C climate change scenarios on AFI simulations, data input were manipulated at each location (2254 grid points) by changing weather data input for AFLA-*maize* model as follow:

i) **temperature**: -6° C, -4° C, -2°C, +2°C, +4°C, +6° C

ii) **precipitation**: -30%, -20%, -10%, +10%, +20%, +30%

iii) **relative humidity**: -30%, -20%, -10%, +10%, +20%, +30%

Temperature changes were applied to daily minimum and maximum temperature data, precipitation changes to the number of rainy days and relative humidity as % variation to the daily average relative humidity, all data input for AFLA-maize model.

Results of the Sensitivity analysis for baseline scenario were shown in Table S1 and were reported as AFI changes (AFI_C) calculated as follow:

$$AFI_C = \frac{AFI_S - AFI_{baseline}}{AFI_{baseline}} \times 100$$

where $AFI_{baseline}$ for wheat is 0.723 and for maize 39.252.

Results are summarised in Table 1S.

Output variations show an extreme variability and an accurate determination of climatic parameters is necessary, especially for temperature.

Parameter modified	Perturbation	AFI Variation (%) Wheat*	AFI Variation (%) Maize*
Temperature (° C)	-6 °	-73.72	-99.63
	-4 °	-51.59	-91.37
	-2 °	-24.17	-56.38
	+2 °	+33.75	+66.42
	+4 °	+104.17	+137.29
	+6 °	+180.94	+194.94
Relative Humidity (%)	-30 %	+0. 24	+0. 35
	-20 %	+0. 24	+0. 35
	-10 %	+ 0.17	+ 0.33
	+10 %	-1.62	-2.91
	+20 %	-7.89	-15.81
	+30 %	-26.52	-48.35
Number of rainy days (%)	-30 %	+7.18	+0.47
	-20 %	+0.88	+0.08
	-10 %	0	+0.01
	+10 %	-3.15	-1.51
	+20 %	-6.34	-3.05
	+30 %	-8.31	-4.42

Table 1S. Relationship between Temperature, Relative Humidity and Number of rainy days perturbation and model output (aflatoxin hazard index, AFI) variation from the reference value (value for average condition without perturbation). Positive changes (increase) for Temperature result in the increase of AFI index, while for the parameters Relative Humidity and Number of rainy days positive changes result in a decrease in AFI.

Accuracy analysis

Based on sensitivity analysis results we used air temperature, relative humidity and rain as regressors in a Linear Regression Model to reconstruct the variability of AFI simulated for the Present Scenario and to estimate uncertainty associated with AFI model when extrapolating the model to climate change scenarios.

The simulations were repeated using air temperature, relative humidity and rain generated from bootstrap samples (10000 iterations; Davison and Hinkley, 1997) for 8 selected years (within the Present Scenario) characterized by the largest variations compared to year representing the mean values. This procedure allows an estimation of AFI simulation accuracy in terms of correlation coefficient (R^2). The results, reported in figure S10, are comparable to the native AFI model accuracy (68%, reported in Battilani et al., 2013); therefore, model accuracy was not affected when extrapolating the model to future climate.

Supplementary Figures (S1-S10)

Figure S.1 Density of pixels (sites) with different AFI values for maize in 3 different climate scenarios, present, +2°C, +5°C. Density distribution was calculated on the basis of the average AFI value computed for maize at harvest using the predictive model AFLA-maize, run in 2254 geo-referenced points throughout Europe, in the 3 scenarios: present (blue solid line), +2 °C (red solid line) and +5 °C (black solid line).

Figure S.2 Density of pixels (sites) with different AFI values for wheat in 3 different climate scenarios, present, +2°C, +5°C. Density distribution was calculated on the basis of the average AFI value computed for wheat at harvest using the modified version of the predictive model AFLA-maize, run in 2254 georeferenced points throughout Europe, in the 3 scenarios: present (blue solid line), +2 °C (red solid line) and +5 °C (black solid line).

Figure S.3 Diagram of the maize AFI values computed using AFLA-maize for each site (pixel) in Europe and for the whole scenario dataset. AFLA-maize was run for 100 years in 2254 geo-referenced points throughout Europe, in the 3 scenarios: (a) present, (b) +2 °C and (c) +5 °C.

Figure S.4 Diagram of the wheat AFI values computed using the modified version of AFLA-maize for each site (pixel) in Europe and for the whole scenario dataset. AFLA-maize was run for 100 years in 2254 georeferenced points throughout Europe, in the 3 scenarios: (a) present, (b) +2 °C and (c) +5 °C.

Figure S.5 Percentage of pixels (sites) where *A. flavus* growth is possible (light blue) and where aflatoxin B_1

contamination was predicted to be above the current legal limit in Europe (pink). AFI was computed for maize (a) and wheat (b) at harvest using AFLA-maize model and its modified version, respectively, run in 2254 geo-referenced points throughout Europe, in the 3 scenarios: present, +2 °C and +5 °C. *Aspergillus flavus* growth is possible when AFI>0 and aflatoxin B_1 contamination is predicted to be above the current legal limit in Europe when AFI≥95 or AFI≥38, respectively for maize and wheat.

Figure S.6 Distribution of median flowering dates of maize over Europe in 3 different climate scenarios, present, +2°C, +5°C. Median flowering dates were based on 100 years in 2254 geo-referenced points throughout Europe, in the 3 scenarios: (a) present, (b) +2 °C and (c) +5 °C; data are reported as percentage of pixels (sites) with flowering in different days of the year (DOY). Percentage of pixels (sites) for each number of days in advance for flowering, resulting for comparison between different scenarios, were also reported: (d) +2 °C *versus* present, (e) +5 °C *versus* present, (f) +2 °C *versus* +5°C.

Figure S.7 Distribution of median harvesting dates of maize over Europe in 3 different climate scenarios, present, +2°C, +5°C. Median harvesting dates were based on 100 years in 2254 geo-referenced points throughout Europe, in the 3 scenarios: (a) present, (b) +2 °C and (c) +5 °C; data are reported as percentage of pixels (sites) with harvesting in different days of the year (DOY). Percentage of pixels (sites) for each number of days in advance for harvesting, resulting for comparison between different scenarios, were also reported: (d) +2 °C *versus* present, (e) +5 °C *versus* present, (f) +2 °C *versus* +5°C.

Figure S.8 Distribution of median flowering dates of wheat over Europe in 3 different climate scenarios, present, +2°C, +5°C. Median flowering dates were based on 100 years in 2254 geo-referenced points throughout Europe, in the 3 scenarios: (a) present, (b) +2 °C and (c) +5 °C; data are reported as percentage

of pixels (sites) with flowering in different days of the year (DOY). Percentage of pixels (sites) for each number of days in advance for flowering, resulting for comparison between different scenarios, were also reported: (d) +2 °C *versus* present, (e) +5 °C *versus* present, (f) +2 °C *versus* +5°C.

Figure S.9 Distribution of median harvesting dates of wheat over Europe in 3 different climate scenarios, present, +2°C, +5°C. Median harvesting dates were based on 100 years in 2254 geo-referenced points throughout Europe, in the 3 scenarios: (a) present, (b) +2 °C and (c) +5 °C; data are reported as percentage of pixels (sites) with harvesting in different days of the year (DOY). Percentage of pixels (sites) for each number of days in advance for harvesting, resulting for comparison between different scenarios, were also reported: (d) +2 °C *versus* present, (e) +5 °C *versus* present, (f) +2 °C *versus* +5°C.

Figure S10. The accuracy of prediction (left) in average AFI for present scenario and 8 selected years, within the present scenario, using linear regression model. Error bars indicate 5% –95% confidence interval based on 10000 bootstrap replicates. In the right panel the Delta T, intended as the difference between the mean air temperature of present scenario and between the mean air temperature of each selected year.

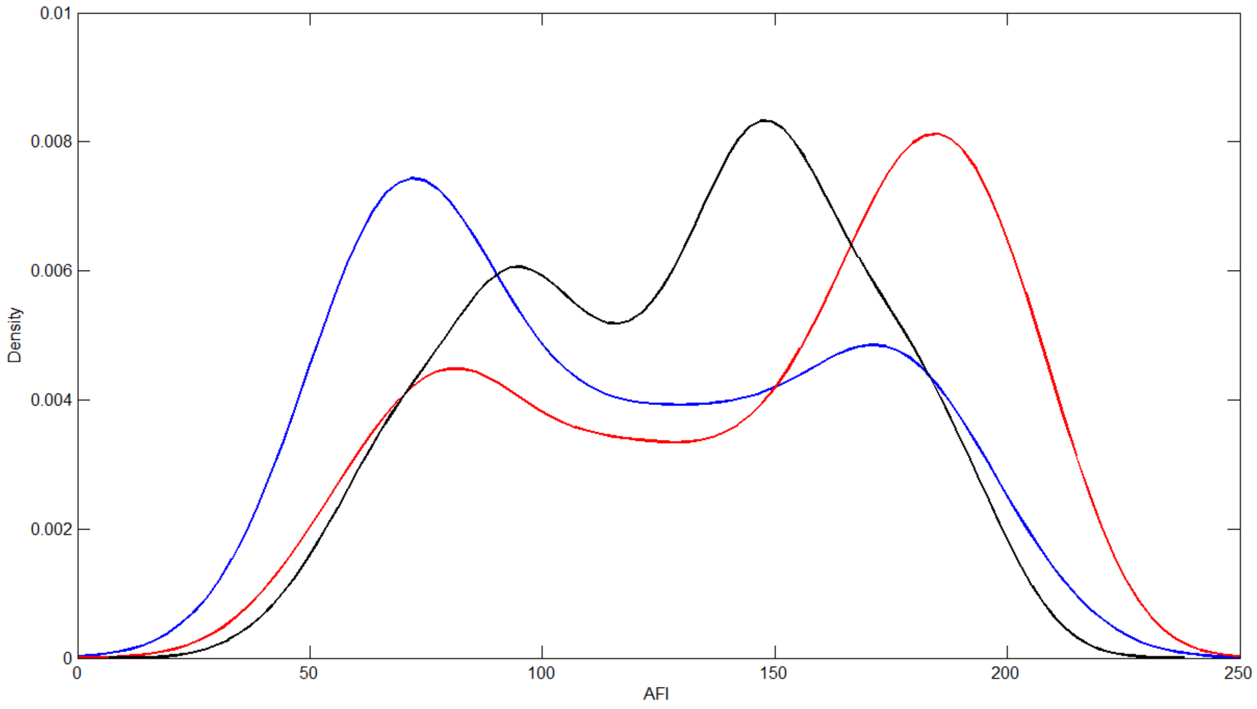

Figure S1

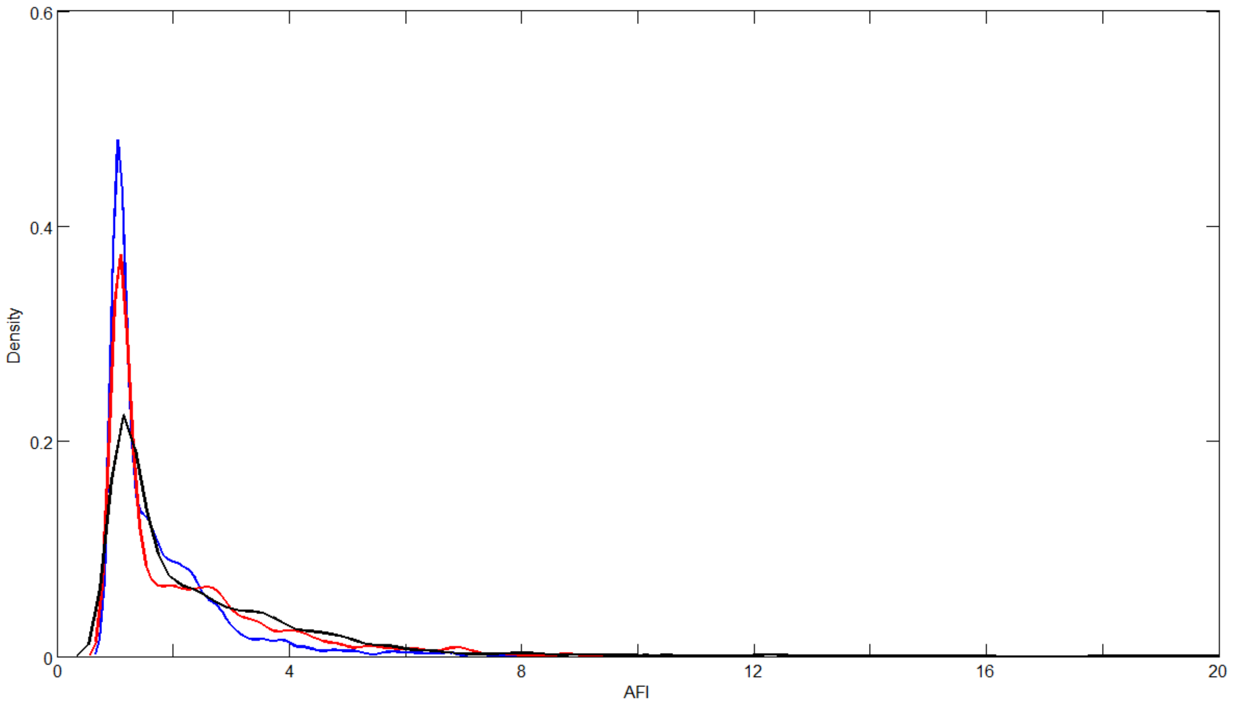

Figure S2

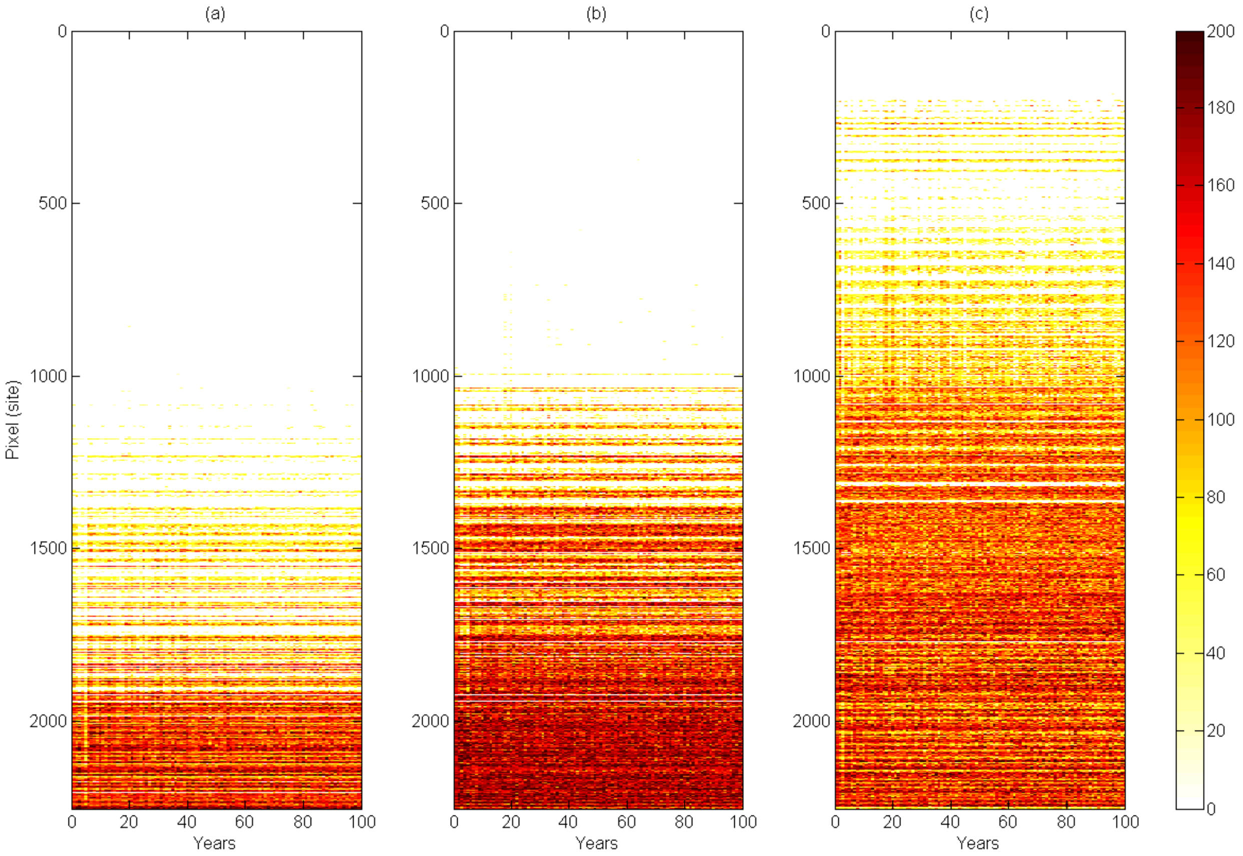

(a)
(b)
(c)
Pixel (site)
0
500
1000
1500
2000
0
20
40
60
80
100
Years
200
180
160
140
120
100
80
60
40
20
0

Figure S3

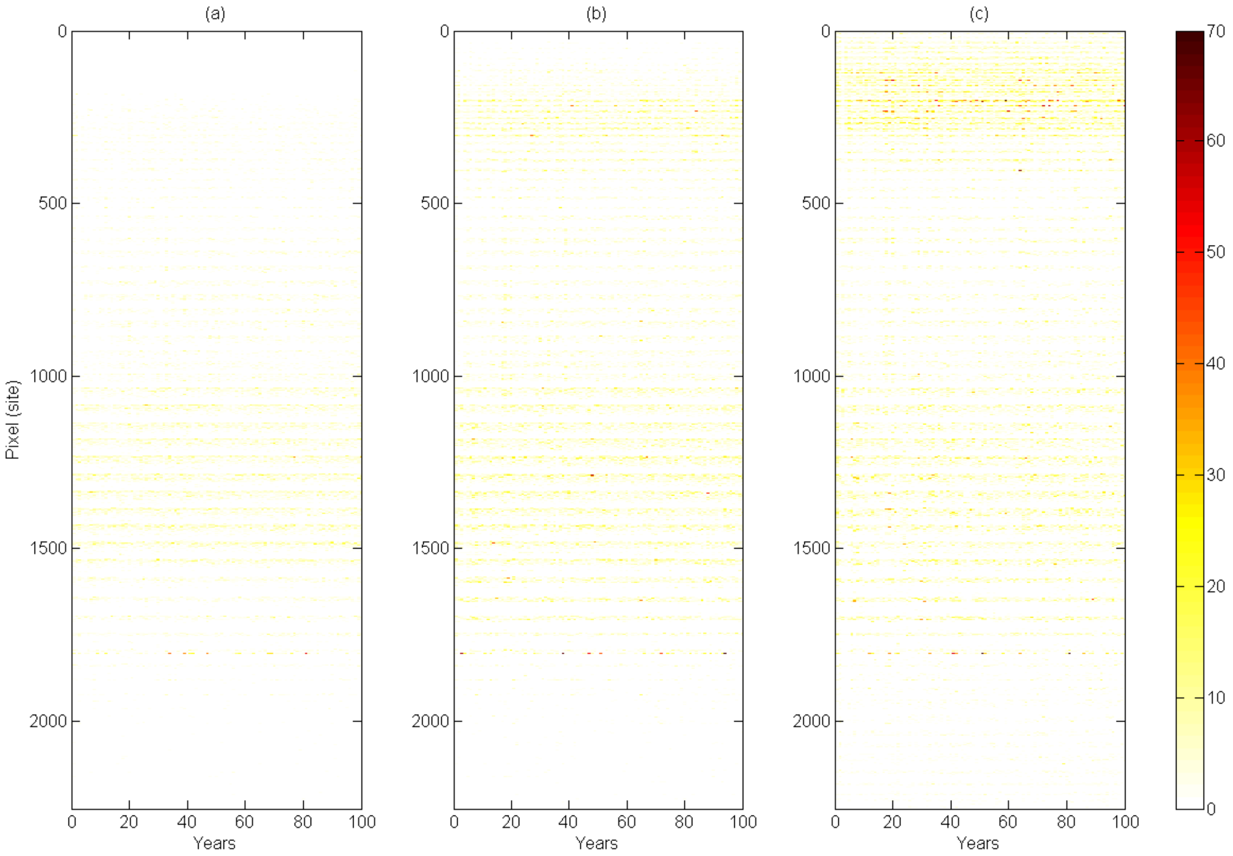

(a)
(b)
(c)
Pixel (site)
Years
Years
Years
0
500
1000
1500
2000
0
20
40
60
80
100
70
60
50
40
30
20
10
0

Figure S4

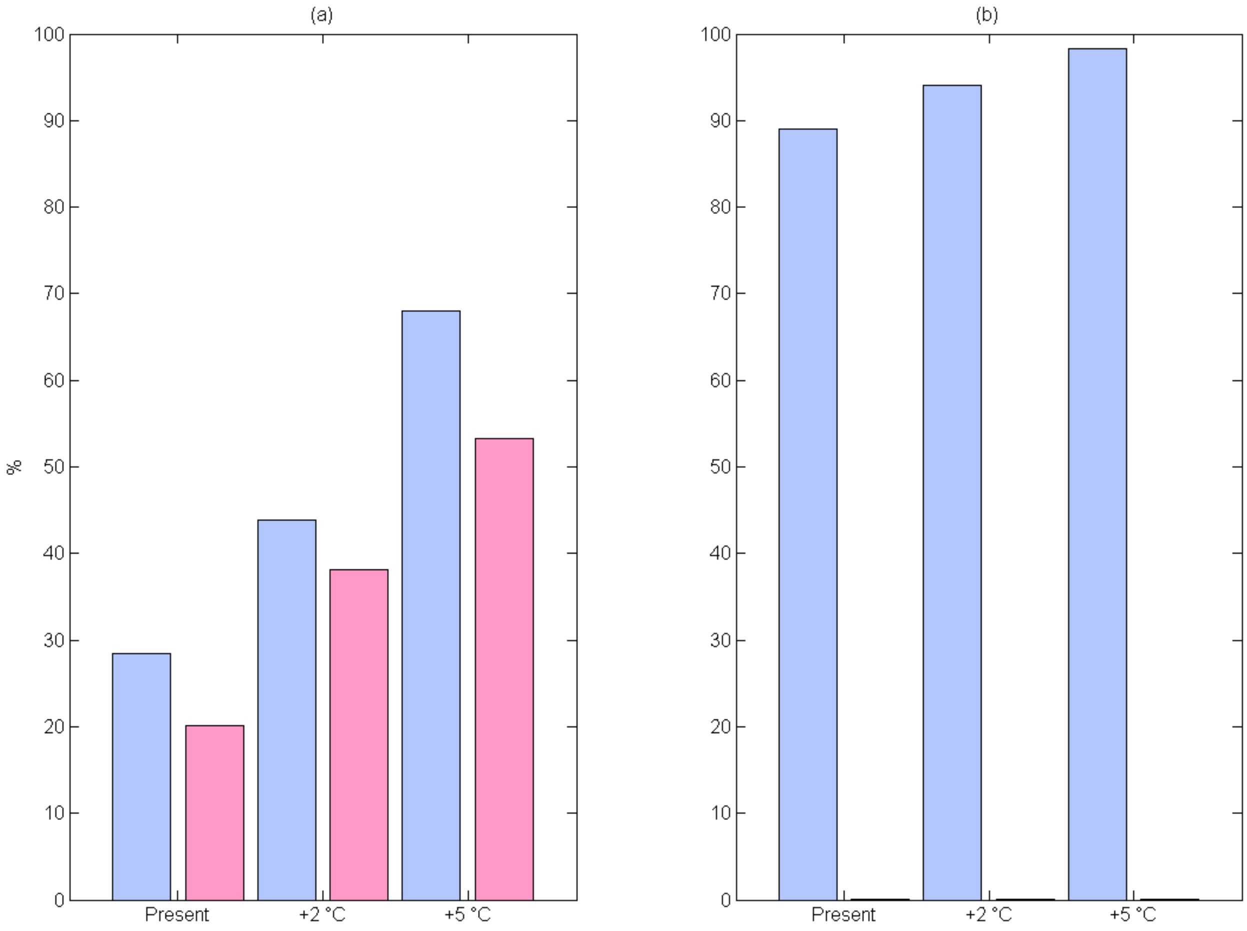

Figure S5

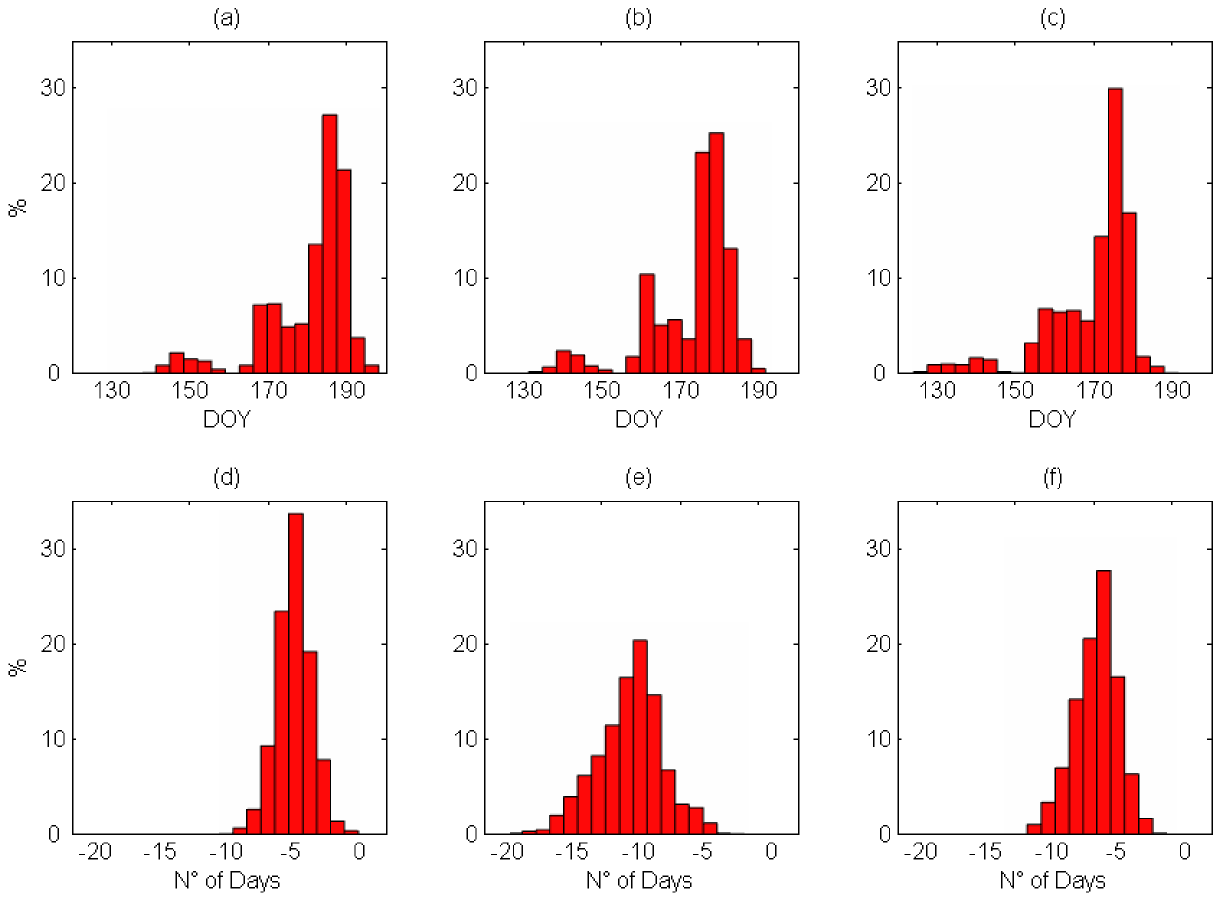

Figure S6

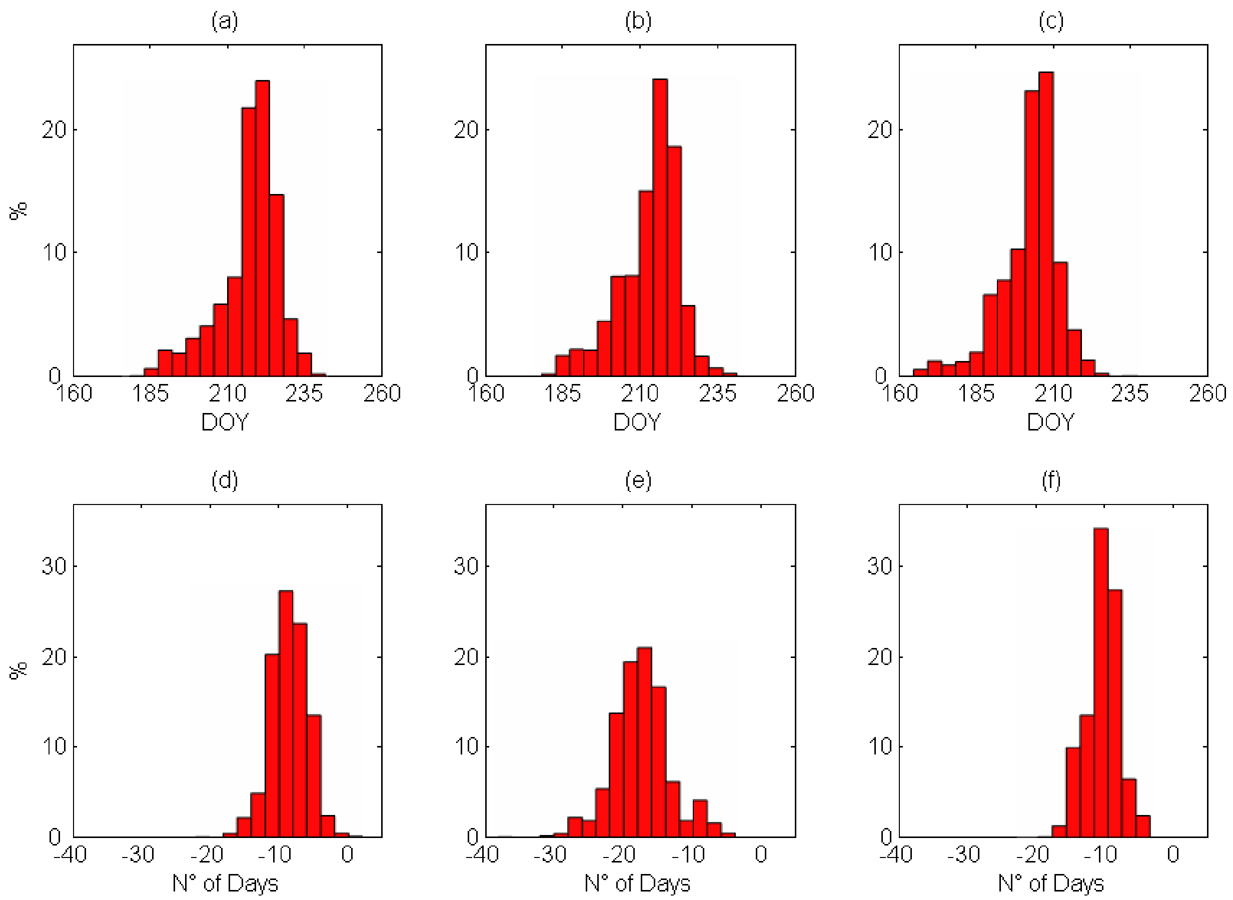

Figure S7

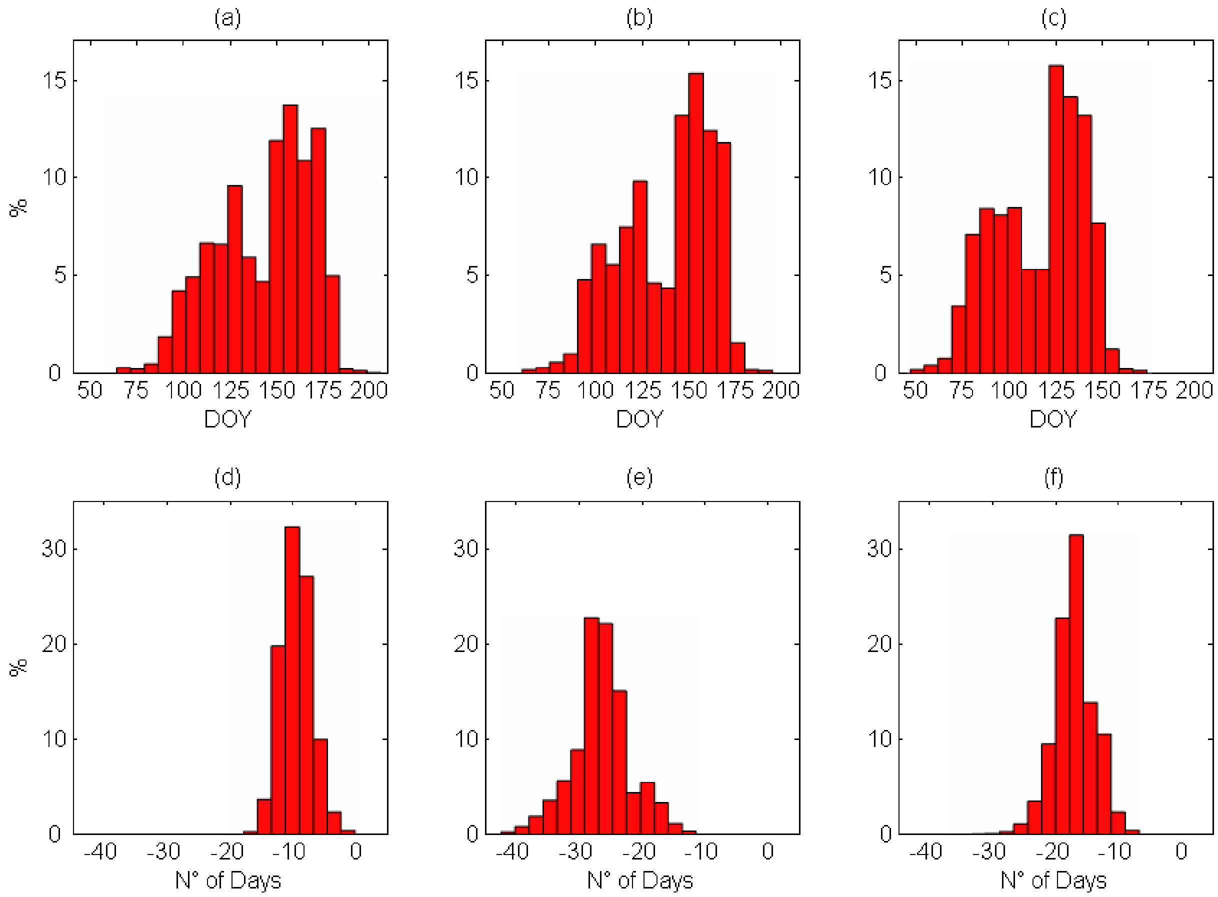
(a)
(b)
(c)
%
15
10
5
0
50 75 100 125 150 175 200
DOY
(d)
(e)
(f)
30
20
10
-40 -30 -20 -10 0
N° of Days

Figure S8

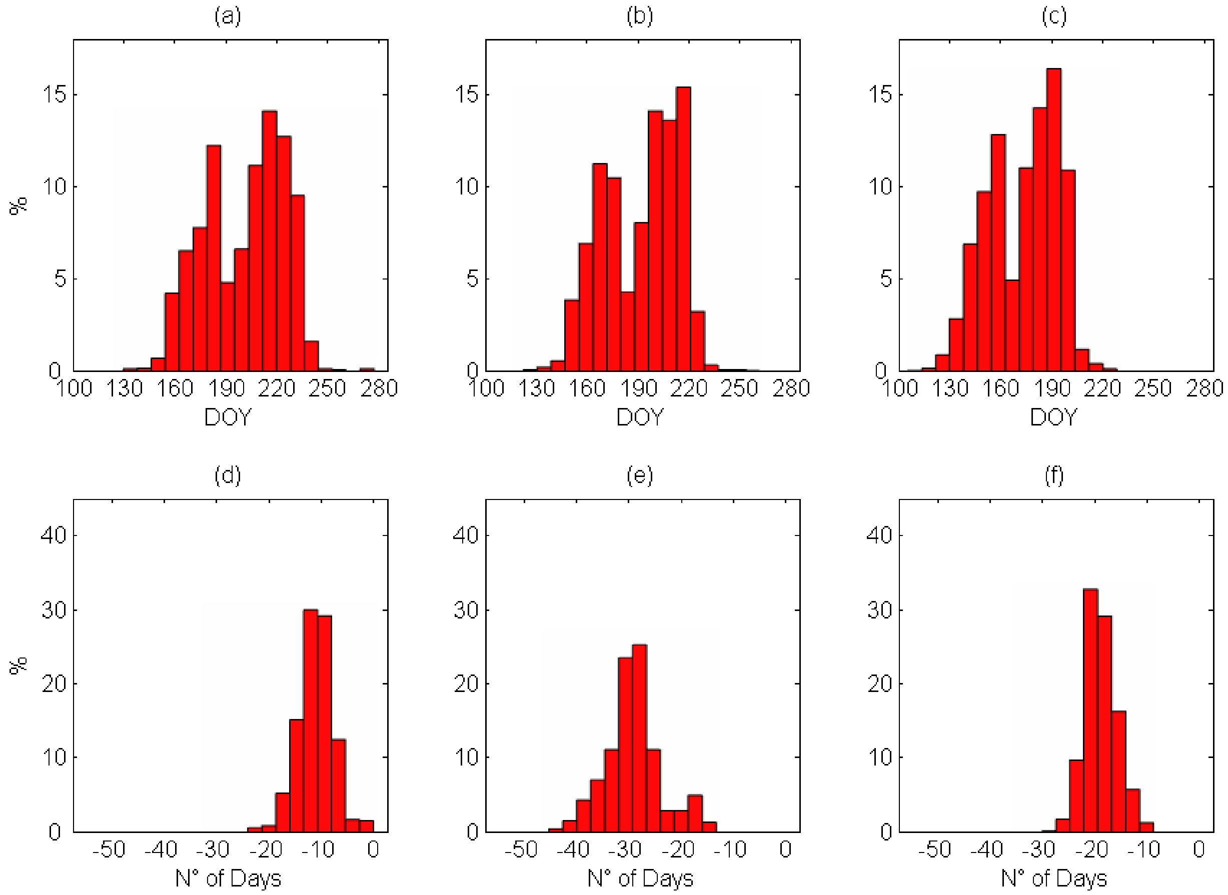
(a)
(b)
(c)
(d)
(e)
(f)
%
DOY
N° of Days
0
5
10
15
20
30
40
100 130 160 190 220 250 280
-50 -40 -30 -20 -10 0

Figure S9

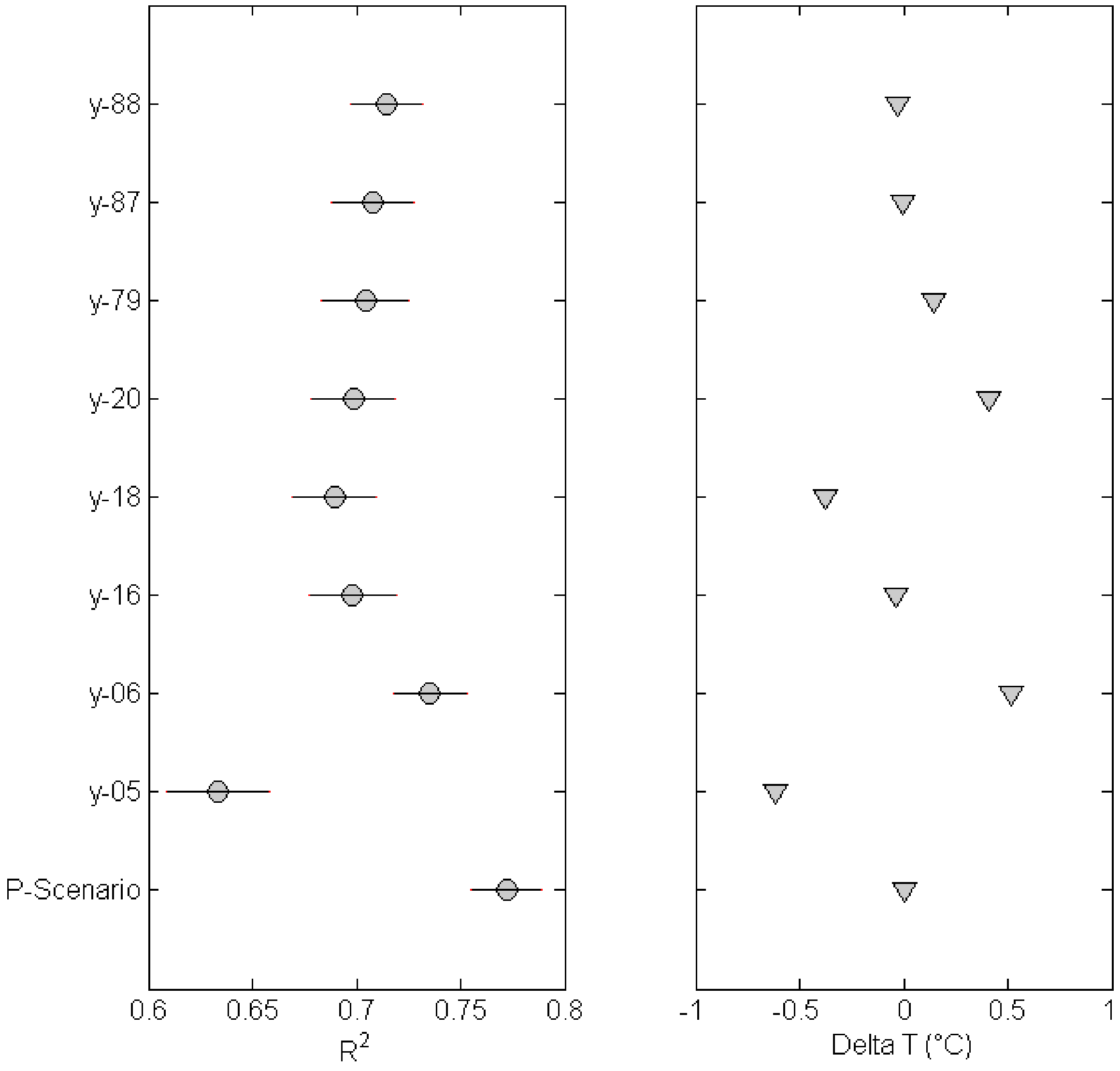
y-88
y-87
y-79
y-20
y-18
y-16
y-06
y-05
P-Scenario
0.6
0.65
0.7
0.75
0.8
R2
-1
-0.5
0
0.5
1
Delta T (°C)

Figure S10
